# Decreased effectiveness of the influenza A(H1N1)pdm09 strain in live attenuated influenza vaccines: an observational bias or a technical challenge?

**DOI:** 10.2807/1560-7917.ES.2016.21.38.30350

**Published:** 2016-09-22

**Authors:** Pasi M Penttinen, Martin H Friede

**Affiliations:** 1 European Centre for Disease Prevention and Control (ECDC), Stockholm, Sweden; 2 Initiative for Vaccine Research (IVR), World Health Organization (WHO), Geneva, Switzerland

**Keywords:** influenza, vaccines and immunisation, vaccine effectiveness

There are currently two types of approved influenza vaccines: inactivated or recombinant vaccines, and live attenuated vaccines. The live attenuated influenza vaccines (LAIV) constructed on a backbone of an A/Leningrad virus strain into which the seasonal haemagglutinin (HA) and neuraminidase (NA) selected for the vaccine were inserted by reassortment, were used in the former Soviet Union for over 50 years [1]. Since the early 2000s, a different attenuated virus strain based on the A/Ann Arbor strain, has been approved for vaccine manufacturing in the United States (US) and more recently in the European Union/European Economic Area (EU/EEA) [2,3]. The proposed advantages of the LAIV were that they had superior efficacy compared to inactivated vaccines in young children [4], they were programmatically more suited to immunisation of children [5] and improved cost-effectiveness could potentially be achieved with childhood LAIV programmes [5-7]. LAIV have also been shown to be of great use in pandemic response since the production yield (doses per egg) is much greater than for inactivated vaccines, and the time between production and release is shorter. In addition, the nasal route of delivery could facilitate rapid population-wide immunisation during pandemics.

The technology to produce pandemic LAIV based on the A/Leningrad backbone has been licensed to the World Health Organization (WHO) for manufacture and use in developing countries. It is estimated that a total production capacity of pandemic LAIV will be ca 500 million doses by 2018 (data not shown). A loss of seasonal LAIV production capacity would impact this pandemic response capacity, and is therefore of global concern.

The US Advisory Committee on Immunization Practices (ACIP) has recently withdrawn the recommendation for use of LAIV in the US for the season 2016/17 following an earlier withdrawal of a preferential recommendation [2]. These decisions were made mainly taking into account the lack of demonstrated vaccine effectiveness (VE) against influenza A(H1N1)pdm09 in observational studies conducted. The studies by the US Centers for Disease Control and Prevention (CDC), and the US Department of Defence, suggested a lower relative effectiveness in comparison to the inactivated influenza vaccine (IIV) [2]. However, two VE studies conducted in Europe and published in this issue of *Eurosurveillance*, reported moderate and reasonable, statistically significant VE in children aged two years and older [8,9]. Furthermore, data from a study funded by the manufacturer of FluMist (US)/Fluenz (Europe) showed similar effectiveness for LAIV in the 2015/16 season [2]. These data were also considered by the ACIP.

In Europe, two EU countries, Finland and the United Kingdom (UK), have introduced LAIV into their publicly-funded routine paediatric vaccination programmes [10]. The two National Immunization Technical Advisory Groups, the UK Joint Committee on Vaccination and Immunisation and the Finnish National Expert Group on Vaccines, considered the available evidence of effectiveness as sufficient to continue the roll-out of vaccination programmes in their countries [11], (personal communication, H Nohynek, September 2016).

Any issues related to LAIV effectiveness or future availability may impact seriously on the roll-out of current and future paediatric and adolescent influenza vaccine and they have potential to affect global pandemic preparedness.

The results from VE studies by Pebody et al. and Nohynek et al. done during the 2015/16 influenza season in the two EU/EEA countries rolling out paediatric and adolescent vaccination programmes including LAIV, document moderate effectiveness of LAIV against influenza A(H1N1)pdm09 in the UK (estimated VE: 41.5%*) and influenza A in Finland (estimated VE: 47.9%) (Table). Results from ongoing analysis of VE studies in Scotland are consistent with these results (personal communication, J McMenamin, September 2016). This contrasts with results from the US CDC studies which found no significant effectiveness against this strain. All the studies showed effectiveness against antigenically matched B viruses (even though numbers of influenza B cases were very low in the Finnish study) and in all of them low level circulation limited assessment of VE against influenza A(H3N2). Each of the studies report a lower effectiveness for LAIV against influenza A(H1N1)pdm09 in comparison with inactivated influenza vaccines, which was not the case in randomised controlled trials when FluMist/Fluenz was authorised.

**Table:** Comparison of study designs and populations assessing vaccine effectiveness of live attenuated influenza vaccine, northern hemisphere countries, United States, United Kingdom and Finland, influenza season 2015/16

	CDC United States	DoD United States	ICICLE United States	PHE United Kingdom	THL Finland
**VE against A(H1N1)pdm09 (95%CI)**	**−21% ** **(−108% to 30%)**	**15% ** (−48% to 51%)*	**50%** (−2% to 75%)*	**41.5%** (−8.5% to 68.5%)*	**47.9% ** **(21.6–65.4%)**
**Study design**	Test-negative case–control	Test-negative case–control	Test-negative case–control	Test-negative case–control	Cohort
**Source population / inclusion criteria**	Children and adolescents aged 2–17 years*	Children and adolescents (Military dependents) aged 2–17 years presenting to participating facilities	Children and adolescents aged 2–17 years	Children and adolescents 2–17 years of age	Children 24–35 months of age
**Inclusion criteria**	MAARI, including cough, and onset of illness ≤ 7 days before enrolment	ILI (fever ≥ 38 °C AND cough and/or sore throat of < 72 hours duration)	ARI with fever ≥ 100.0°F (37.8 °C), duration < 5 days	ILI	Laboratory-confirmed influenza
**Assessment of vaccination status**	Current-season vaccination (at least one vaccine dose ≥ 14 days before illness onset; vaccine records obtained from electronic medical records and immunisation registries for children aged 2–8 years; with addition of reported vaccination for patients aged 9–17 years)*	Electronic medical records	Vaccination status was ascertained by medical record review and/or state or regional vaccine registries	Self-reported by patients to general practitioners	National immunisation registry
**Case definition**	RT-PCR-positive subjects*	RT-PCR-positive subjects	RT-PCR positive subjects	RT-PCR positive subjects	RT-PCR, multiplex RT-PCR, culture and/or antigen detection test
Final sample size (number of vaccinated with LAIV / number of non-vaccinated)*	133/1,078*	93/338*	101/594	111/514*	8,323/46,119
**Adjusted for**	Study site, age, self-rated general health status, race/hispanic ethnicity, interval (days) from onset to enrolment, and calendar time	Age groups, three time periods	Site, age group, visit date, outpatient visits in past 6 months, health insurance, and sex	Age group, sex, month, pilot area and surveillance scheme	Propensity scores, and adjusted by their quintiles
**Source**	ACIP presentation 22 June 2016 also cited in [2] and personal communication (J Clippard, September 2016)*	ACIP presentation 22 June 2016 also cited in [2] and personal communication (S Federinko, September 2016)*	ACIP presentation 22 June 2016 also cited in [2] and personal communication (H Caspard, September 2016)*	Pebody 2016 [9]	Nohynek 2016 [8]

ACIP: Advisory Committee on Immunization Practices; ARI: acute respiratory infection; CDC: Centers for Disease Control and Prevention; DoD: Department of Defence; ICICLE: Influenza Vaccine Effectiveness Influenza Clinical Investigation for Children; ILI: influenza-like illness; MAARI: medically attended acute respiratory infection; PHE: Public Health England; THL: Terveyden ja hyvinvoinnin laitos (National Institute for Health and Welfare).

All studies, with the exception of the Finnish one, use the test-negative case–control study methodology which has the potential to control for many of the biases inherent with observational studies (Table) but lacks power when stratifying e.g. in strata with small sample sizes. This methodology was extensively evaluated in the past and can be considered the gold standard for observational VE studies [12-16]. Therefore the observed discrepancies between the conducted studies are surprising and deserve careful assessment.

Potential explanations for the discrepancies in the VE study results for LAIV during the 2015/16 influenza season could be related to study design, analytical methods to calculate the adjusted VE, or true differences in effectiveness due to properties of the virus or the target populations. Methodological and analytical differences should affect the effectiveness results for influenza B viruses and inactivated influenza vaccines in the same way. All of the studies agree on some LAIV effectiveness against B viruses. LAIV used in Europe and North America are produced in the same factory, therefore it is unlikely that differences in the composition of the vaccine explain the differences in VE.

The factors driving the lower effectiveness observed in the US over the past five years compared to that seen in the European studies are likely to be related to population or programme-specific effects. In this regard, the comparatively high coverage of influenza vaccination in children 6 months to 2 years of age in the US, before the age at which LAIV is given as part of the vaccination programme, may be a contributing factor. Other factors could include environmental issues such as storage and administration temperature particularly since an early formulation of this vaccine was shown to be thermolabile [17].

Nonetheless, a lower comparative (compared to IIV) effectiveness against the influenza A(H1N1) strains was observed in all the studies. The comparatively lower effectiveness is most likely related to the biological properties of the influenza A(H1N1)pdm09 strain used in the vaccines. Potential explanations include (i) the transition to quadrivalent formulations which occurred 5 years ago, and a potential competition between the B strains and the A(H1N1)pdm09 strain and (ii) a lower fitness of the A(H1N1)pdm09 strain in terms of sialic acid binding specificity, rate of cell entry, replication and budding.

Following the ACIP decision, the European Centre for Disease Prevention and Control (ECDC) and WHO have facilitated a series of discussions between relevant public health research groups in order to review available data and generate hypotheses to explain the differences in VE results and to develop a framework to test these hypotheses. To complement this, WHO organised a global consultation in Geneva on 20–21 September 2016 to discuss potential explanations for recent evidence of decreased performance of LAIV compared with IIV. At this meeting, the potential explanations outlined above were discussed and apart from the methodological constraints of observational studies, they were considered to be likely but requiring research to confirm. Gathering more data, testing the hypotheses and identifying corrective actions will require dedicated resources The manufacturer of the LAIV used in Europe and North America has embarked on a comprehensive virological research programme to study many of these hypotheses to improve and optimise the effectiveness of the 2017/18 vaccine formulation (personal communication, M Downham, 20 September 2016). The involved public health agencies are seeking to enhance their VE studies and have embarked upon better understanding drivers of the variability in the effectiveness estimates. Unfortunately, additional national or supranational funding sources do not appear to be available to rapidly fund adequately scaled operational public health research during the upcoming 2016/17 season.

The US Vaccines for Children Programme had ordered 14 million doses of LAIV for the upcoming 2016/17 influenza season, representing roughly two thirds of the global sales for 2016 [18]. They will now not be used due to the June ACIP decision. Difficult commercial decisions will now need to be taken in the coming months regarding the production for the 2017/18 northern hemisphere season. In a situation where all influenza vaccines used in Europe are produced by commercial manufacturers EU/EEA countries depend on commercial decisions by the manufacturers for availability of LAIV for continued immunisation programmes.

In addition to the LAIV currently used in Europe and North America, several manufacturers in developing countries have started the production of LAIV using the A/Leningrad backbone, and one Indian manufacturer produces pandemic and nationally approved seasonal LAIV vaccines. No data regarding the 2015/16 VE are available from these manufacturers. The policy decisions made in Europe and in the US have an impact on commercial decisions by all manufacturers and as mentioned above, on the global capacity to respond to influenza.

The US Food and Drug Authority (FDA) and the European Medicines Agency (EMA) consider that the benefit–risk ratio of the LAIVs licenced by them remains positive and no changes in market authorisation are envisaged [17]. In the coming months, EMA will introduce a new guideline requiring manufacturers to provide annual VE estimates as part of the market authorisation [19].

The VE results for LAIV 2015/16 clearly show the necessity of assessing VE on an annual basis. With core funding from ECDC, the European Influenza Monitoring Vaccine Effectiveness (I–MOVE) network has established a methodology and an EU/EEA-wide network to estimate seasonal VE [20]. The challenge of conducting these studies is to find study sites with sufficiently high uptake of influenza vaccines and the resources to recruit large enough sample sizes. The European Innovative Medicines Initiative has called for a proposal to prepare for a platform to enable these studies, in particular to establish a governance model where such studies could be undertaken in a public-private partnership. Such partnership should include public health agencies recommending and assessing vaccination programmes and manufacturers producing the vaccines in an atmosphere of transparency and scientific independence [21].

The European seasonal influenza immunisation programmes of children are based on estimated healthcare cost savings (Finland) [7] and estimated reductions of transmission of influenza and indirect protection of the elderly and risk groups (UK) [22]. Both programmes are currently being rolled out, especially in the UK, in a step-wise fashion. Therefore full assessments of the impact of these programmes are only awaited within the next few years. Now these programmes are faced with two immediate risks, before such assessments can be made; on the one hand a low (or non-existent as in the US) effectiveness which would decrease the impact of the programmes and on the other hand the dependence on the commercial decisions of the manufacturers.

Virological, epidemiological and immunological studies are urgently needed to understand the reasons behind the decrease of the influenza A(H1N1)pdm09 component of LAIV to inform the vaccine strain selection decision for the northern hemisphere in February 2017, the public health decisions on the vaccines to be recommended for the 2017/18 season and to support sound commercial decisions by the vaccine manufacturers.

## *Author’s correction

The VE for 2-17 year-olds in the UK was corrected on request of the authors on 22 and 29 September 2016. In addition, figures for the final sample sizes for CDC, DoD and PHE and case definition for CDC were corrected in the Table on 29 September 2016.

Following publication, the exact confidence intervals for VE in DoD and ICICLE were provided to the authors in personal communications and specified in the Table on 29 September 2016. Exact age groups for the source population and information on vaccination status in the CDC study were provided to the authors in personal communications and specified in the Table on 29 September 2016.
