# Authors’ correction for Euro Surveill. 2016;21(38)

**DOI:** 10.2807/1560-7917.ES.2016.21.39.30357

**Published:** 2016-09-29

**Authors:** Eurosurveillance editorial team

**Affiliations:** 1 European Centre for Disease Prevention and Control (ECDC), Stockholm, Sweden

In the article ‘Decreased effectiveness of the influenza A(H1N1)pdm09 strain in live attenuated influenza vaccines: an observational bias or a technical challenge?' by Penttinen and Friede, published on 22 September 2016, the following corrections were made post publication on request of the authors:

• VE for 2–17 year-olds in the UK was corrected on request of the authors on 22 and 29 September 2016. 

• Figures for the final sample sizes for CDC, DoD and PHE and case definition for CDC were corrected in the Table on 29 September 2016. 

• The exact confidence intervals for VE in DoD and ICICLE were specified in the Table, and exact age groups for the source population and information on vaccination status in the CDC study were specified in the Table on 29 September 2016.

